# A Novel Untargeted Molecular Detection Technique for Rapid Fecal Microbiota Profiling in Very Preterm Infants: Optimization, Genus‐Level Comparison, and Application

**DOI:** 10.1096/fj.202502006RR

**Published:** 2025-11-03

**Authors:** R. R. de Kroon, A. J. van Wesemael, A. H. van Kaam, P. H. M. Savelkoul, M. Boon, A. E. Budding, H. J. Niemarkt, T. G. J. de Meij

**Affiliations:** ^1^ Department of Pediatric Gastroenterology Emma Children's Hospital, Amsterdam UMC Amsterdam the Netherlands; ^2^ Amsterdam Reproduction and Development Research Institute, Amsterdam UMC Amsterdam the Netherlands; ^3^ Amsterdam Gastroenterology Endocrinology Metabolism Research Institute, Amsterdam UMC Amsterdam the Netherlands; ^4^ Neonatal Intensive Care Unit Emma Children's Hospital, Amsterdam UMC Amsterdam the Netherlands; ^5^ Medical Microbiology, Infectious Diseases and Infection Prevention Maastricht University Medical Center Maastricht the Netherlands; ^6^ inBiome BV Amsterdam the Netherlands; ^7^ Neonatal Intensive Care Unit Máxima Medical Centre Veldhoven the Netherlands; ^8^ Department of Electrical Engineering Technical University Eindhoven Eindhoven the Netherlands

**Keywords:** 16S rRNA gene sequencing, clinical application, fecal microbiota profiling, gastrointestinal microbiota, IS‐pro, nanopore sequencing, preterm infants

## Abstract

Gut microbiota profiling shows potential for improving care in the neonatal intensive care unit (NICU). However, common techniques, including 16S rRNA gene and metagenomic sequencing, have limited bedside applicability. The IS‐pro microbiota assay provides species‐level abundances within 5 h. We aimed to optimize the taxa annotation for preterm infants (phase 1), compare its findings to 16S sequencing on the genus level (phase 2), and apply the assay in a preterm cohort (phase 3). 1445 fecal samples from 479 preterm infants (24–30 weeks gestation) across 10 NICUs were analyzed with IS‐pro. For phase 1 (optimization), IS‐pro amplicons of 32 fecal samples were additionally analyzed with nanopore sequencing to expand the IS‐pro matching database. For phase 2 (comparison), 41 samples were compared to 16S sequencing. In phase 3 (application), the optimized IS‐pro assay was applied to the total cohort. Following phase 1, a mean relative abundance of 82.5% was successfully annotated. In phase 2, IS‐pro showed high concordance with 16S sequencing, with a strong positive correlation between the two techniques (Pearson's correlation coefficient: 0.77, SD 0.24). In phase 3, IS‐pro analysis of the full cohort revealed *Staphylococcus, Klebsiella, Enterococcus, Escherichia‐Shigella*, and *Streptococcus* as the predominant genera in the first 4 weeks of life. Our findings demonstrate that the IS‐pro microbiota assay effectively detects and quantifies key bacterial taxa in fecal samples of preterm infants, with outcomes highly concordant with 16S sequencing. Unlike traditional techniques, IS‐pro is a rapid tool, illustrating its potential for clinical practice. Future studies should explore its applications in the NICU.

## Graphical abstract

This study optimized the matching database of the IS‐pro microbiota assay for very preterm infant fecal samples, compared the output with commonly used 16S rRNA gene sequencing, and applied the technique to a cohort of 479 very preterm infants. Our findings demonstrate that the IS‐pro assay effectively detects and quantifies key bacterial taxa in very preterm infants, with outcomes highly concordant with 16S rRNA gene sequencing. It is a rapid technique (< 5 h) with potential for application in clinical practice (Created in Biorender. Amsterdam UMC, Eminds (2025), https://BioRender.com/b8mn6x5).

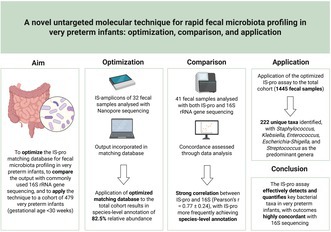

**Abbreviations**
16S: 16S rRNA gene sequencing
AA: absolute abundance
FAFV: *Firmicutes, Actinobacteria, Fusobacteria*, and *Verrucomicrobia*
GA: gestational age
GI: gastrointestinal
IS: interspace
LOS: late‐onset sepsis
NEC: necrotizing enterocolitis
NICU: neonatal intensive care unit
PCR: polymerase chain reaction
PERMANOVA: permutational multivariate analysis of variance
RA: relative abundance
RFU: relative fluorescent units

## 1 Introduction

In the past decades, the role of the gut microbiome in human health has been extensively studied. The gut microbiome is a complex community of microorganisms, including bacteria, fungi and viruses, residing in the gastrointestinal (GI) tract [1]. Alterations in the bacterial microbiota are associated with a variety of conditions, including inflammatory bowel disease, obesity, diabetes, cardiovascular disease, sepsis, and neurological disorders [2, 3, 4, 5, 6, 7, 8]. Clinical applications of microbiota analyses may be particularly promising for the advancement of neonatal care for preterm infants (gestational age (GA) < 37 weeks). Preterm infants have an altered microbiota development, which is associated with important short‐term morbidities, such as necrotizing enterocolitis (NEC) and late‐onset sepsis (LOS), and long‐term outcomes [9, 10, 11, 12]. Preclinical alterations in the gut microbiota of infants with NEC and LOS have previously been identified [13, 14, 15, 16]. Detection of these early intestinal microbiota changes, associated with NEC or LOS, may facilitate early diagnosis, which is often challenging due to nonspecific clinical presentation resulting in delayed initiation of treatment. Early identification of high‐risk infants through microbiota profiling may provide a critical window of opportunity for clinicians to initiate targeted interventions aimed at preventing disease progression.

As the majority of the bacterial species colonizing the gut are highly refractory to culture‐based techniques, gut microbiota research advanced rapidly with the development of culture‐independent techniques in the 1990s. To date, 16S rRNA gene sequencing is the most frequently utilized method in microbiota research [17, 18]. While 16S sequencing facilitates large‐scale microbiota analyses, it has its limitations, including the inability to differentiate bacterial species with closely related sequences. Therefore, it typically provides genus‐level identification rather than species‐level identification [19]. To overcome this limitation, metagenomic sequencing is increasingly adopted. This technique allows for species‐ and strain‐level characterization, while also revealing the microbial functional profile [18]. Despite their role in advancing microbiota research, both methods face challenges in clinical applicability due to high costs, non‐standardized bioinformatic workflows, and long turnaround times from sampling to sequencing results (1.5–7 working days) [20, 21].

To implement microbiota profiling in clinical practice, an alternative, rapid, standardized microbiota testing method is preferred. Such a microbiota‐based technique should be capable of detecting a wide spectrum of clinically relevant bacteria in neonatal fecal samples. Achieving this requires robust DNA extraction and bacterial detection methods with a standardized bioinformatics pipeline for taxonomic classification, ensuring consistent results across laboratories and technicians [22]. Additionally, a rapid microbiota‐based test should have a short turnaround time (ideally within hours to a day) and be easily integrated into clinical workflows. The molecular microbiota detection technique IS‐pro (inBiome, Amsterdam, the Netherlands) provides an alternative for high‐resolution bacterial profiling, with the potential for implementation in clinical practice. IS‐pro is a PCR‐based molecular technique that combines length polymorphisms of the 16S to 23S interspace rDNA with phylum‐specific fluorescently labeled primers. It generates absolute (AA) and relative abundances (RA) with species‐level accuracy within 5 h following sampling [13, 23, 24, 25]. The output of the IS‐pro assay is incompatible with frequently used sequencing reference databases, such as Reengines or Silva. Therefore, an in‐house matching database is employed to assign bacterial species to IS‐pro output. IS‐pro is currently predominantly used to characterize the bacterial composition of physiologically sterile bodily samples, such as blood and synovial aspirates [26, 27].

Studies using the IS‐pro assay have also been conducted on the vaginal [28] and intestinal microbiota of adults and older children [24]. However, preterm infants have a distinct intestinal microbiota composition [9]. Although the matching database, used for the interpretation of IS‐profiles, is extensive, we hypothesized that the pre‐existing matching database used for IS‐pro may not be fully suitable to assess the very preterm microbiota composition. Furthermore, the comparability to the most utilized microbiota profiling technique, 16S sequencing, has yet to be assessed for these sample types. Therefore, the current study aimed to optimize, compare, and apply the use of IS‐pro in 1445 fecal samples from 479 very preterm infants admitted to one of ten participating NICUs in the Netherlands and Belgium. We optimized the existing matching database (phase 1), compared IS‐pro to 16S sequencing outcomes with the improved database (phase 2), and applied the IS‐pro assay on our total cohort of fecal samples derived from very preterm infants (phase 3).

## 2 Methods

### 2.1 Study Design

**FIGURE 1:**
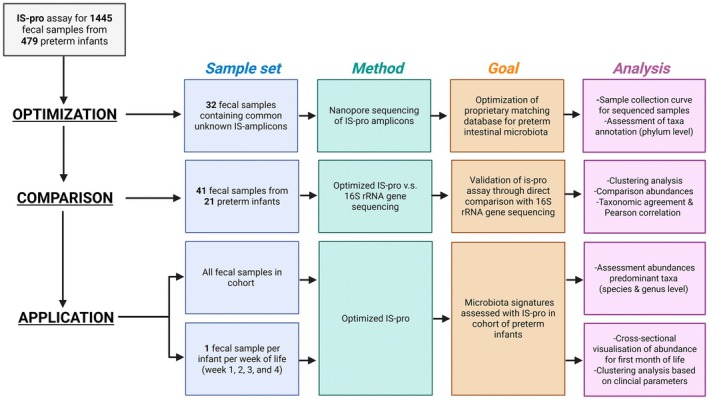
Schematic overview of the current study. This study is conducted in three phases: (1) optimization, (2) comparison, and (3) application. 1445 fecal samples from 479 preterm infants were analyzed by the IS‐pro microbiota assay (InBiome, Amsterdam, the Netherlands). In phase 1 (optimization), the IS‐pro amplicons of a subset of fecal samples were assessed with nanopore sequencing to refine the proprietary matching database for a preterm population. In phase 2 (comparison), a subset of fecal samples was analyzed using 16S rRNA gene sequencing, and the fecal microbiota signatures were compared with those from IS‐pro, using the matching database optimized in phase 1, to assess the technique comparability. Finally, in phase 3 (application), we examined the fecal microbiota signatures of the total preterm cohort, using the optimized database. Created in Biorender. Amsterdam UMC, Eminds (2025). https://BioRender.com/vpfqggf.

This study is embedded in an ongoing, multicenter, national, cohort study. The aim of this overarching study is the development of novel microbiota‐based biomarkers for the prediction of NEC and LOS. Clinical data and fecal samples of preterm infants (GA 24–30 weeks between October 2014 and March 2021, GA 24–28 weeks from March 2021 onwards) born at one of ten NICUs in the Netherlands and Belgium are collected daily for the first 29 days of life. Study approval was provided by the local institutional board of all participating centers (2014.386 (A2020.190)). Written informed consent was obtained from parents or legal caretakers. For the current study, all fecal samples analyzed by IS‐pro between October 2023 and October 2024 were included (Figure 1). This included infants born between January 2016 and October 2023. The study was conducted in three phases: (1) optimization, (2) comparison, and (3) application.

### 2.2 Fecal Sample and Clinical Data Collection

Fecal samples were collected directly from the diapers by NICU nurses and stored at −20°C within 1 h after collection. Samples collected after March 2021 were moved to long‐term storage at −80°C until processing. Demographic and clinical data were extracted from the electronic patient files and stored in Castor EDC (2021.5).

### 2.3 IS‐Pro Microbiota Assay

Fecal microbiota analysis was performed using the IS‐pro microbiota assay (inBiome, Amsterdam, the Netherlands) [13, 23, 24, 25]. IS‐pro discriminates bacterial species based on the length of the 16S‐23S rDNA interspace (IS) region by targeting phylum‐specific sequence variations. The IS‐pro assay consists of three steps: (1) DNA isolation, (2) two polymerase chain reactions (PCRs), and (3) capillary electrophoresis.

#### 2.3.1 DNA Isolation

DNA was isolated from 100 to 400 mg fecal sample with the IVD‐labeled NucliSENSeasyMag extraction machine (Biomérieux, Marcy l'Etoile, France), as described by the manufacturer. 250 μL Bacterial Shock Buffer 1 (0.2 M NaOH, 1% (w/v) SDS) was added to the feces, followed by vortexing (~15 s) and incubation (~10 min) in a thermomixer (95°C at 800 RPM, Thermomixer comfort, Eppendorf, Hamburg, Germany). 25 μL Bacterial Shock Buffer 2 (1 M Tris–HCl, pH 7.4) was added prior to vortexing (~15 s). After centrifugation at 18.000 RCF (~2 min), the supernatant was added to the EasyMAG vessel, filled with 1 mL EasyMag lysis buffer and 1 mL EasyMag AL buffer. Following incubation for 10 min at room temperature, 70 μL magnetic silica beads, as provided in the EasyMAG DNA extraction kit, were added and the solution was resuspended. The DNA extraction was performed on the easyMAG machine, according to the Specific A protocol, and the DNA was eluted in 110 μL elution volume. Each DNA isolation run included one negative control sample. The DNA was stored at 4°C until further processing.

#### 2.3.2 Polymerase Chain Reaction

The IS‐fragments are amplified from the bacterial DNA in two separate PCRs with proprietary phylum‐specific fluorescent primers. The lengths of the amplified IS‐fragments are species‐specific and are used to identify bacteria with species‐level accuracy. The first PCR contains two fluorescently labeled PCR primers. The first primer amplifies the phylum Bacteroidetes and the second primer amplifies the phyla Firmicutes, Actinobacteria, Fusobacteria, and Verrucomicrobia (FAFV) (FIRBAC Mastermix 2.0, inBiome, Amsterdam, the Netherlands). The second PCR contains a single fluorescently labeled PCR primer, which amplifies the phylum Proteobacteria, an internal amplification control, and human DNA (PROTEO+IC Mastermix 2.0, inBiome). For the first PCR, 10 μL isolated DNA was mixed with 15 μL FIRBAC Mastermix 2.0, and for the second PCR, 10 μL isolated DNA was mixed with 15 μL PROTEO Mastermix 2.0. The solution was centrifuged at 1000 RPM (~30 s). Amplification was done with a GeneAMP PCR system 9700 (Applied Biosystems, Foster City, CA, USA). The program started with 10 min at 95°C, followed by 35 cycles of 94°C for 30 s, 56°C for 45 s, and 72°C for 1 min. Final elongation was 2 min at 72°C. Each PCR plate contained a negative and positive control (POSMAT, inBiome). The PCR product was stored at 4°C until further processing.

#### 2.3.3 Capillary Electrophoresis

Following the PCRs, the amplified fluorescently labeled IS‐fragments are separated and detected by capillary electrophoresis. 20 μL eMix (inbiome) was added to 2.5 μL of the FIRBAC PCR product and the PROTEO‐IC PCR product in a single well. After spinning down, the 96‐well plate was heated at 94°C for 3 min, followed by cooling to 25°C. DNA fragment analysis was performed by the ABI Prism 3500(XL) Genetic Analyzer (Applied Biosystems, Foster City, CA, USA), according to the manufacturer's instructions. The fragments are separated by size based on their total charge. The dye signals of fragments become fluorescent by crossing a laser beam and can be detected by a camera. The fluorescence signal is then converted into digital data compatible with software applications, as described below.

### 2.4 Data Processing

The ABI Prism 3500XL data was converted into IS‐profiles using Antoni Lab Cloud software, which denoises data and aligns the IS‐fragments to taxa by means of a proprietary matching database (inBiome, Amsterdam, The Netherlands). The IS‐profile of a sample contains peaks based on three parameters: (1) length of the IS‐fragments, measured in nucleotides, (2) abundance of each IS‐fragment, measured in relative fluorescent units (RFUs), and (3) the fluorescent color of each IS‐fragment, representing the phylum group of the fragment (e.g., Bacteroidetes, FAFV, or Proteobacteria). All IS‐fragments that were successfully annotated to a taxon were included for analysis. For IS‐fragments that could not be annotated, a cut‐off value of > 4000 RFU was used. PCR and capillary electrophoresis were repeated for samples that lacked the internal amplification control and for plates containing contaminated negative controls and/or inadequate positive controls. If contamination persisted, all samples from the same DNA extraction run were repeated.

### 2.5 Optimization of IS‐Pro for Fecal Microbiota Profiling in Preterm Infants

In phase 1 (optimization), the IS‐amplicons from a subset of samples were assessed with long‐read nanopore sequencing to refine the proprietary matching database for microbiota profiling in a preterm population. The matching database has been crafted and validated for sterile samples, such as blood, plasma, or synovial fluid, containing over 350 species. However, we hypothesized that the database may not be optimal for preterm fecal microbiota profiling. To optimize the database for preterm infants, fecal samples containing IS‐fragments unassignable to known bacterial species with the existing database were selected based on the presence of one or more of the 10 most common unknown IS‐fragments within that sample. Approximately three samples per unknown IS‐fragment were assessed by means of nanopore sequencing of the IS‐pro amplicons (Appendix S1), resulting in a selection of 32 fecal samples for nanopore sequencing. Novel taxa that were identified by sequencing were added to the matching database. Before and after optimization of the matching database, the mean RA for the known and unknown bacterial taxa was assessed, to determine whether the database was deemed sufficient for microbiota profiling in preterm infants.

### 2.6 Comparison of IS‐Pro to 16S rRNA Gene Sequencing for Fecal Microbiota Profiling in Preterm Infants

A total of 41 fecal samples from 21 unique infants in our cohort had previously undergone 16S sequencing (Appendix S1), as part of a separate study in a different laboratory, and were included in phase 2 (Comparison). The comparability of the methods was assessed using principal coordinate analysis (PCoA) based on Bray–Curtis dissimilarity of genus‐level RAs. A scatter plot representation of the mean RA by 16S sequencing and IS‐pro was generated including the genera that could be annotated by both techniques. At the sample level, the taxonomic agreement on the genus level was assessed for the two methods, as well as the correlation between 16S sequencing and IS‐pro by means of the Pearson correlation coefficient.

### 2.7 Application of IS‐Pro for Fecal Microbial Profiling in Preterm Infants

In phase 3 (Application), we examined 1445 fecal samples by IS‐pro in our very preterm cohort. We assessed the mean RA on genus level and species level in the total cohort. For a subset of our total cohort, we cross‐sectionally assessed the course of the mean RA on genus level in week 1, 2, 3, and 4 of life. A maximum of one sample per infant per week available from the total cohort was included in the analysis, to prevent skewing of the results. Additionally, beta‐diversity differences on species level for common drivers of the preterm gut microbiota (gestational age, birthweight, center of birth, mode of delivery, and week of life, respectively) were visualized in a PCoA plot.

### 2.8 Statistical Analysis

Bray–Curtis dissimilarity calculation was performed in R studio version 4.2.1. using the vegan package. Homogeneity of multivariate dispersion was assessed with the betadisper function, followed by permutation tests for dispersion. All PCoA plots based on beta‐diversity differences using Bray–Curtis distances were tested for significance using permutational multivariate analysis of variance (PERMANOVA). A *p* value ≤ 0.05 was considered significant.

## 3 Results

### 3.1 Baseline Characteristics of Preterm Population

1445 fecal samples from 479 preterm infants were included in our cohort. Table 1 displays demographic and clinical characteristics of study participants.

**TABLE 1:** Demographic and clinical characteristics of the very preterm cohort.

	Infants (*n* = 479)
Gestational age in weeks (mean [SD in days])	26.7 (±10)
Birth weight in grams (mean [SD])	921 (±230)
Biological sex, female (*n* [%])	235 (49)
Vaginal mode of delivery (*n* [%])	236 (49)
Singleton gestation (*n* [%])	335 (70)
Apgar score 5 min postpartum (median [IQR])	7 (2)
Surfactant administration (*n* [%])	307 (64)
Probiotic administration in the first 29 days of life (*n* [%])	166 (35)
Antibiotic administration in the first 29 days of life (*n* [%])	441 (92)
Ratio antibiotic administration first 29 days of life (median [IQR])	0.28 (0.38)
29‐day mortality (*n* [%])	18 (4)

### 3.2 Optimization of IS‐Pro for Fecal Microbiota Profiling in Preterm Infants

In phase 1 (Optimization), a subset of fecal samples (*n* = 32) was analyzed using long‐read nanopore sequencing of IS‐pro amplicons to enhance the proprietary matching database for the preterm intestinal microbiota. The fecal samples were selected based on whether they contained at least one of the 10 most prevalent unknown IS‐fragments in our cohort. Figure S1 displays the collector's curve for the sequenced fecal samples. A total of 54 unique bacterial taxa were identified in this sequencing effort. The samples contained a total of 76 unknown IS‐fragments, of which 36 were successfully annotated following nanopore sequencing. When using this optimized matching database in the total cohort (*n* = 1445 fecal samples), a mean RA of 82.5% could be annotated to the species level, compared to 80.4% prior to optimization. The remaining 17.5% unassigned RA was primarily composed of Proteobacteria (43%), FAFV (30%), and Bacteroidetes (27%).

### 3.3 Comparison of IS‐Pro to 16S rRNA Gene Sequencing for Fecal Microbiota Profiling in Preterm Infants

**FIGURE 2:**
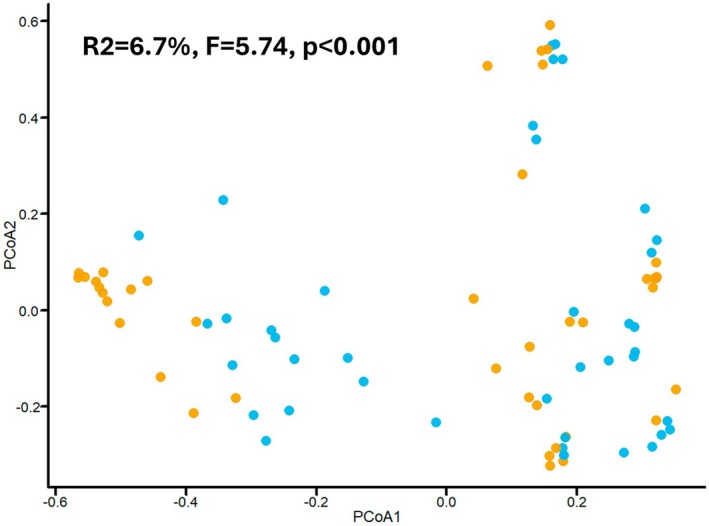
The 16S rRNA gene sequencing and IS‐pro methods explained approximately 7% of the variation in beta‐diversity based on Bray–Curtis dissimilarity, generating highly similar results. Bacterial beta‐diversity as assessed by principal coordinate analysis (PCoA) based on Bray–Curtis dissimilarity is displayed using data aggregated on genus level. Potential clustering based on microbiome composition is assessed for a subset of fecal samples (*n* = 41 samples from 21 unique infants). Coloring is based on the used analysis method; orange displays all samples assessed by 16S sequencing and blue displays all samples assessed by IS‐pro. Statistical analysis was performed by PERMANOVA. *R* ^2^, *F* value, and *p*‐statistic are displayed in the figure. Additionally, homogeneity of dispersion was assessed using the betadisper function in the vegan package (permutational analysis of multivariate dispersions (PERMDISP); *F* = 12.2, *p* = 0.001). A *p* value below 0.05 was considered significant. PCoA, principal component analysis, PERMANOVA, permutational multivariate analysis of variance; PERMDISP, permutational analysis of multivariate dispersions.

In phase 2 (Comparison), a subset of fecal samples (*n* = 41) was analyzed by both 16S sequencing and IS‐pro, using the database optimized in phase 1, to assess the comparability of the two methods. A small portion of the variation observed in microbiota profiles could be attributed to the sequencing method itself (*R* ^2^ = 6.7%, *F* = 5.74, *p* **≤** 0.001; Figure 2), whereas the dominant sources of variation were individual sample identity (*R* ^2^ = 79.4%, *F* = 3.95, *p* **≤** 0.001) and patient identity (*R* ^2^ = 69.5%, *F* = 6.95, *p* **≤** 0.001; Figure S2). The two techniques differ in their ability to classify bacterial taxa. With 16S sequencing, on average, 68.6% of the total bacterial abundance per sample could be resolved to the genus level, and 31.0% to the species level, while less than 1% remained unclassified at the genus level. In comparison, IS‐pro could resolve 81.5% of the bacterial signal to the species level. The remaining 18.5% could only be classified at higher taxonomic levels, namely at the phylum or phylum‐group level (31% assigned to the FAFV group, 33% to Proteobacteria, and 36% to Bacteroidetes).

**FIGURE 3:**
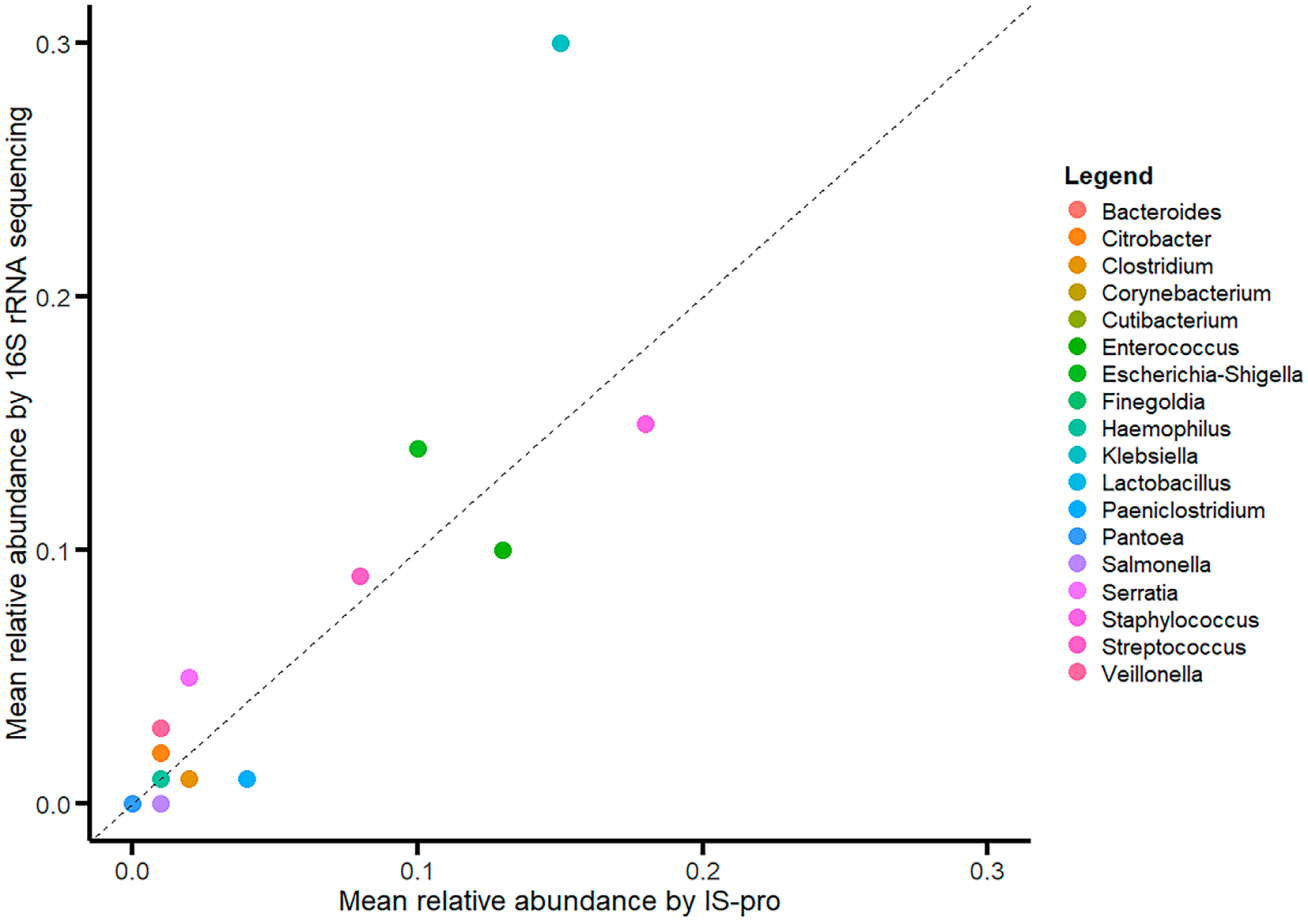
Comparable mean relative abundance on genus level for 16S rRNA gene sequencing and IS‐pro. The scatterplot displays the mean relative abundance (RA) displayed as a proportion, of the bacterial genera that are detected by both 16S rRNA gene sequencing and IS‐pro in a subset of preterm fecal stool samples (*n* = 41 stool samples from 21 preterm infants). Each genus is displayed by a different color, depicted in the legend. A reference line is included. The closer the genus is placed to the black dashed reference line, the more similar the mean RA is between the two techniques. For all bacterial genera, the mean RA over the two methods is similar, except for *Klebsiella,* which has a higher mean RA when assessed with 16S sequencing in comparison to IS‐pro. RA, relative abundance.

Next, we evaluated the taxonomic agreement between the two methods. Among all samples, 27 genera with an average RA greater than 1% were detected by 16S sequencing, and 24 genera were detected by IS‐pro; 16 genera were identified by both methods. The majority of the genera that were detected by a single method had a low abundance (mean RA < 1%), except for three genera identified solely by 16S sequencing: *Anaerococcus* (mean RA 1.1%), *Ligilactobacillus* (mean RA 1.9%), and *Parabacteroides* (mean RA 1.3%). At the genus level, the overall RA distributions were comparable between the two methods, except for *Klebsiella*, which appeared more abundant in 16S profiles than in IS‐pro profiles (Figure 3).

At the individual sample level, a median of 2 genera (range: 1–8) was detected by 16S sequencing, compared to a median of 3 genera (range: 2–7) detected by IS‐pro. Across samples, the median number of genera detected by both methods was 2 (range: 1–4). When expressed relative to the number of genera detected by 16S sequencing, the median proportion of shared genera per sample was 75% (Table S1). When assessing the correlation at the sample level by means of the Pearson's correlation coefficient, we found a strong positive correlation with a mean coefficient of 0.77 (SD 0.24). Based on our findings, microbiota profiling of preterm fecal samples by the IS‐pro showed high concordance with 16S sequencing.

### 3.4 Application of IS‐Pro for Fecal Microbiota Profiling in Preterm Infants

**FIGURE 4:**
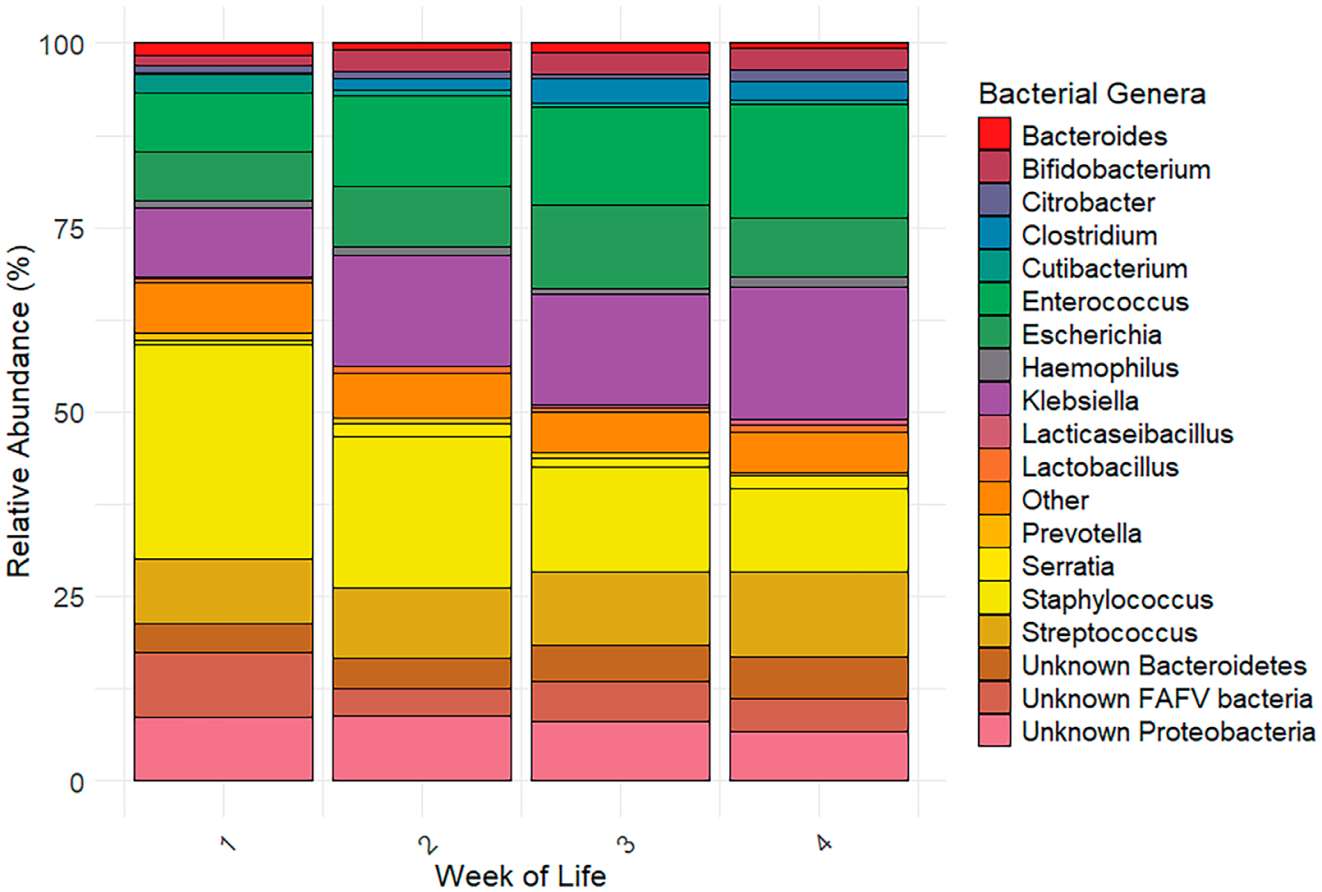
Decreased *Staphylococcus* and increased *Klebsiella* and *Enterococcus* in the fecal samples of preterm infants during the first month of life analyzed with IS‐pro. This figure displays a stacked bar chart depicting the median relative abundance (RA) (%) of bacterial genera in preterm fecal samples for the first week of life. Each bar represents the samples collected in 1 week, respectively, week 1 (postnatal day 0–7, *n* = 256 samples), week 2 (postnatal day 8–14, *n* = 296 samples), week 3 (postnatal day 15–21, *n* = 233 samples) and week 4 (postnatal day 22–29, *n* = 167 samples). The height of each segment within the bar corresponds to the median RA of a specific bacterial genus. Bacterial genera are color‐coded for clarity. The cumulative contribution sums to 100% for each week. A maximum of one sample per infant per week available from the total cohort was included in the analysis, to prevent skewing of the results. The infants that are included in week 1 only overlap partly with the infants that are included in week 2, 3, and 4, and vice versa. RA, relative abundance.

Finally, we assessed the fecal microbiota signatures in our preterm cohort. In all 1445 fecal samples combined, 222 unique bacterial taxa could be identified. Seventy‐five bacterial taxa were identified in ≥ 1% of the fecal samples. Table 2 displays an overview of the 10 most prevalent genera and 12 most prevalent species that were identified in the preterm fecal samples, with their corresponding mean RA. Next, we cross‐sectionally assessed the microbiota signatures in the first month of life (Figure 4). Visual inspection demonstrated a decrease in *Staphylococcus* over the course of the first 29 days of life, while *Klebsiella* and *Enterococcus* appear to increase. Finally, we assessed whether there was evident clustering based on common drivers of the preterm gut microbiota (e.g., GA, birth weight, center of birth, delivery mode, week of life, and biological sex, respectively). Center of birth and week of life significantly affected beta‐diversity (Figure S3). However, the variance explained is low for both parameters (*R* ^2^ = 0.3%, *p* = 0.024; *R* ^2^ = 3.0%, *p* **≤** 0.001, respectively).

**TABLE 2:** Overview of the most prevalent bacterial genera and species or equivalence sets in fecal samples of preterm infants as assessed with IS‐pro.

	Mean relative abundance (%) (SD; minimum, maximum)
Predominant bacterial genera	
*Staphylococcus*	20.2 (23.9; 0, 95.3)
*Klebsiella*	13.8 (19.4; 0, 83.1)
*Enterococcus*	12.2 (17.8; 0, 96.5)
*Escherichia‐Shigella*	9.5 (16.5; 0, 88.3)
*Streptococcus*	9.1 (12.9; 0, 96.6)
*Bifidobacterium*	2.5 (5.7; 0, 45.0)
*Clostridium*	2.0 (7.5; 0, 49.4)
*Bacteroides*	1.5 (6.8; 0, 71.4)
*Serratia*	1.4 (6.5; 0, 82.2)
*Citrobacter*	1.1 (5.3; 0, 61.1)
Predominant bacterial species	
*Enterococcus faecalis*	11.1 (17.5; 0, 96.5)
*Staphylococcus epidermidis*	10.0 (18.1; 0, 95.3)
*Escherichia coli/Shigella* spp.	9.5 (16.6; 0, 93.4)
*Klebsiella pneumoniae* complex*/Enterobacter cloacae* complex	8.8 (16.2; 0, 81.6)
*Staphylococcus haemolyticus*	6.1 (17.8; 0, 95.5)
*Streptococcus bovis* group/ *Streptococcus intermedius*	5.6 (10.7; 0, 83.6)
*Klebsiella aerogenes/oxytoca*	5.0 (11.8; 0, 73.0)
*Staphylococcus aureus*	2.5 (8.2; 0, 72.1)
*Clostridium perfringens*	1.5 (6.3; 0, 49.4)
*Serratia marcescens*	1.3 (6.5; 0, 82.6)
*Streptococcus* sp.	1.3 (4.9; 0, 56.4)
*Bifidobacterium longum* subsp. *infantis*	1.2 (3.6; 0, 23.1)

*Note:* This table depicts the mean relative abundance (RA) of the most prevalent bacterial genera and species in the total cohort (*n* = 1445 stool samples from 479 preterm infants), as assessed with IS‐pro. Only the genera and species ≥ 1% mean RA were included. With the IS‐pro assay, the majority of bacterial taxa is annotated to a single species. However, 29 equivalence sets have been identified, consisting of multiple possible bacterial species, which cannot be discriminated from each other by the IS‐pro assay. Four equivalence sets were present in ≥ 1% mean RA in the total cohort*: Escherichia coli/Shigella, Klebsiella pneumoniae/Enterobacter cloacae* complex, *Streptococcus bovis/Streptococcus intermedius, and Klebsiella aerogenes/oxytoca* (9.5, 8.8, 5.6, and 5.0 mean RA, respectively).

Abbreviation: RA, relative abundance.

## 4 Discussion

In the current study, we optimized, compared, and applied the IS‐pro assay, a molecular technique for fecal microbiota profiling in very preterm infants (GA 24–30 weeks), using nanopore‐ and 16S sequencing. Our results demonstrate high concordance between the IS‐pro assay and the optimized matching database and 16S sequencing on the genus level. Additionally, we evaluated the outcomes of IS‐pro based on the assessment of 1445 fecal samples from 479 very preterm infants, demonstrating that IS‐pro is a useful technique for fecal microbiota profiling in very preterm infants in a clinical setting.

In phase 1 (Optimization), we utilized long‐read nanopore sequencing of the IS‐fragments to optimize the existing proprietary database. Given that microbial communities can vary significantly between environments, incorporation of environment‐specific taxonomic profiles may further improve annotation accuracy for distinct populations, such as very preterm infants [29]. This optimization led to the identification of 47% of yet unknown IS‐fragments within the sequenced dataset, resulting in a 2% increase in the RA of identifiable bacterial species. We were able to identify the majority of IS‐fragments, resulting in a mean RA of 82.5% that could be annotated to the species level. The remaining mean 17.5%, which remained unclassified at the species level, could be assigned to phylum or phylum‐group level. Future use of IS‐pro for preterm fecal microbiota profiling should consider that some, potentially uncommon bacterial taxa, are still absent from the current database and may contribute to the residual unclassified fraction.

In phase 2 (Comparison), we found minimal clustering explained by the sequencing method, when assessing the beta‐diversity with PCoA based on Bray–Curtis dissimilarity (*R* ^2^ = 6.7%, *F* = 5.74, *p* **≤** 0.001), indicating that IS‐pro and 16S sequencing generate highly comparable profiles. When assessing all samples together, we found that the predominant genera were the same for both methods (*Staphylococcus, Streptococcus, Enterococcus, Klebsiella*, and *Escherichia/Shigella*, respectively) and the yielded RA was comparable between IS‐pro and 16S sequencing. For *Klebsiella*, whilst the detection rate was similar, the RA was higher for 16S sequencing compared to IS‐pro. Although IS‐pro is limited by a relatively large proportion of taxa that were only attributed to a phylum or phylum‐group (mean RA 19% for IS‐pro compared to < 1% for 16S in this subset), the taxa can be annotated to the species level more frequently (81% for IS‐pro compared to 31% for 16S), providing greater taxonomic precision. Noteworthy, a small number of low abundance taxa were exclusively identified by one method, demonstrating a difference in detection sensitivity or potential misinterpretation of artifacts or noise as true taxa.

On sample level, we found a strong positive correlation between the two techniques (Pearson's correlation coefficient 0.77, SD 0.24). Additionally, IS‐pro identified a median of 1 additional genus per sample. This underestimation of the taxa found by 16S sequencing is commonly described in literature comparing 16S sequencing with other next‐generation sequencing approaches [30, 31]. The inter‐method differences, such as the difference in *Klebsiella* abundance, and a strong, but not perfect correlation, could be explained by multiple factors, such as different DNA extraction protocols, PCR primer bias, and the different computational pipeline and database used for taxonomic assignment. Multiple studies show that differences in DNA extraction protocols, different computational pipelines and databases can cause varying and even weak concordance between 16S sequencing procedures [32, 33]. This context further underscores the high comparability observed between IS‐pro and 16S sequencing, despite the notable differences between the two techniques. We argue that IS‐pro is a good alternative to 16S sequencing, especially for standardized rapid testing in clinical practice and when taxonomic classification at the species level is preferred compared to higher taxonomic ranks. However, for in depth microbiota studies, 16S sequencing or metagenomic sequencing should still be considered.

In phase 3 (Application), we found that the most frequently identified genera in our cohort were attributed to the FAFV phylum group, followed by the Proteobacteria and Bacteroidetes phyla, respectively. These findings are in line with previous studies in which the same phylum distribution of the preterm gut microbiota is observed [34, 35]. The predominant bacterial genera in our cohort, as assessed by IS‐pro, were *Staphylococcus, Streptococcus, Enterococcus, Klebsiella*, and *Escherichia/Shigella*. Our results are consistent with other studies that have identified facultative anaerobic bacteria as common early colonizers of the preterm gut [34, 36]. Other commonly identified genera within our cohort, such as *Bifidobacterium, Clostridium*, and *Bacteroides*, may also be of clinical importance as these have been linked to health outcomes [9, 10, 13, 36, 37]. The ability of IS‐pro to detect these clinically relevant genera and species is promising for its application in clinical practice, as it may facilitate microbiome‐based diagnostics and interventions. Lastly, we examined changes in gut microbiota composition over the first 29 days of life. The dominant genera identified at baseline remained prevalent across all time points. We observed an increasing RA of facultative anaerobes such as *Klebsiella* and *Enterococcus*, alongside a decrease in *Staphylococcus*. The observed shifts are consistent with the natural succession of the neonatal gut microbiota, transitioning from early colonization by skin‐ and environment‐associated taxa to the enrichment of gut‐adapted facultative and eventually strict anaerobic communities [38, 39].

This study has several strengths and limitations. Strengths include the large multicenter dataset of preterm stool samples. We designed our study to optimize a preterm‐specific matching database, to accommodate the detection of the most common bacterial taxa found in preterm stool samples. By analyzing a subset of our larger preterm cohort with 16S sequencing, we were able to directly compare the IS‐pro outcomes to 16S sequencing. Lastly, the setup of our study allowed for application of the IS‐pro assay by assessing the physiological development of the preterm gut microbiome in the first month of life. Limitations for our study include the small subset of samples for comparison with 16S sequencing and the small range in gestational age of preterm infants (24–30 weeks). A further expansion of the IS‐pro matching database is likely required to expand our approach to more mature preterm or term populations, which may differ in general microbiota composition. Additionally, while species‐level IS‐pro data were available, the majority of data were presented at the genus level to align with the resolution of 16S, but it inherently limits the taxonomic depth of our findings.

In conclusion, we optimized, compared, and applied the IS‐pro assay for fecal microbiota profiling in very preterm infants. Our findings demonstrate that IS‐pro is a reliable method, highly concordant with 16S sequencing, suitable for the detection of the most prevalent bacterial genera in this unique population. While IS‐pro will unlikely replace 16S sequencing or metagenomic sequencing in a research setting, it is a rapid standardized tool, making it suitable as a diagnostic tool in clinical practice. Future studies should focus on assessing the feasibility of this technique for clinical implementation, while also addressing the key challenges prior to its widespread adoption in daily clinical practice.

## Author Contributions

**R. R. de Kroon:** conceptualization, data curation, formal analysis, methodology, project administration, visualization, and writing – original draft preparation. **A. J. van Wesemael:** conceptualization, data curation, formal analysis, methodology, project administration, visualization, and writing – original draft preparation. **A. H. van Kaam:** supervision and writing – review and editing; **P. H. M. Savelkoul:** writing – review and editing. **M. Boon:** data curation and writing – review and editing. **A. E. Budding:** conceptualization, data curation, and writing – review and editing. **H. J. Niemarkt:** conceptualization, supervision, and writing – review and editing. **T. G. J. de Meij:** conceptualization, supervision, and writing – review and editing.

## Conflicts of Interest

A.E.B. is CEO of inbiome bv. P.H.M.S. is co‐founder and shareholder of inbiome bv. M.B. is employed by inbiome bv. The other authors declare no conflicts of interest.

## Supporting information


**Appendix S1:** fsb271207‐sup‐0001‐AppendixS1.docx.

**Figure S1:** Following optimization of the IS‐pro matching database for preterm fecal microbiota profiling, more than 90% mean relative abundance per phylum was successfully annotated to species‐level. Collector's curve for subset of fecal samples (*n* = 32) assessed by nanopore sequencing for optimization of matching database for preterm population. The number of unique bacterial taxa (*Y*‐axis) is plotted against the cumulative sample size (*X*‐axis) to assess the taxa richness and sampling sufficiency.
**Figure S2:** Sample identification and patient identification explains approximately 80% and 70%, respectively, of beta‐diversity differences based on Bray–Curtis dissimilarity. Bacterial beta‐diversity as assessed by principal coordinate analysis (PCoA) based on Bray–Curtis dissimilarity is displayed. Potential clustering based on microbiome composition is assessed for a subset of fecal samples (*n* = 41 samples from 21 infants). (S1A) Coloring in plot is based on sample identification (*R* ^2^ = 79.4%, *F* = 3.95, *p* ≤ 0.001), regardless of bacterial profiling technique; each color represents a specific fecal sample. (S1B) Coloring in plot is based on patient identification (*R* ^2^ = 69.5%, *F* = 6.95, *p* < 0.001; PERMDISP: *F* = 1.7, *p* = 0.07), regardless of bacterial profiling technique; each color represents an individual patient. Statistical analysis was performed by PERMANOVA. Additionally, homogeneity of dispersion was assessed using the betadisper function in the vegan package. *R* ^2^, *F* value, and *p*‐statistic are displayed for each figure, as well as *F* value and *p* value for the permutational analysis of multivariate dispersions (PERMDISP). A *p* value ≤ 0.05 was considered significant. The largest variation is explained by week of life. Abbreviations: PCoA, principal component analysis, PERMANOVA, permutational multivariate analysis of variance; PERMDISP, permutational analysis of multivariate dispersions.
**Table S1:** Taxonomic agreement between 16S rRNA gene sequencing and IS‐pro on sample level. A subset of fecal samples (*n* = 41 samples from 21 preterm infants) was assessed by both IS‐pro and 16S rRNA gene sequencing (16S). The 16S‐IS‐pro‐agreement was computed per sample. The median number and range of genera detected per sample for 16S sequencing and IS‐pro, as well as the median and range of shared genera per sample is displayed. Lastly, the percentage per sample of common genera divided by the total found genera with 16S and IS‐pro respectively, is depicted. Abbreviations: 16S, 16S rRNA gene sequencing.
**Figure S3:** Minimal differences in bacterial beta‐diversity in 1445 fecal samples of preterm infants explained by clinical parameters as analyzed with IS‐pro. Bacterial beta‐diversity as assessed by principal component analysis (PCoA) based on Bray–Curtis dissimilarity is displayed. Clustering based on microbiome composition was assessed for all fecal samples. Clustering based on the following clinical parameters was assessed: gestational age (1: 24–26 weeks of gestation, 2: 26–28 weeks of gestation, and 3: 28–30 weeks of gestation; *R* ^2^ = 0.03%, *F* = 1.94, *p* = 0.061; PERMDISP: *F* = 1.3, *p* = 0.285), birth weight (1: < 1000 g, 2: ≥ 1000 g; *R* ^2^ = 0.01%, *F* = 1.51, *p* = 0.160; PERMDISP: *F* = 1.8, *p* = 0.183), center of birth (center 1 until 10; *R* ^2^ = 0.3%, *F* = 2.48, *p* = 0.024; PERMDISP: *F* = 2.7, *p* = 0.002), delivery mode (0: vaginal delivery, 1: cesarean section; *R* ^2^ = 0.9%, *F* = 7.20, *p* ≤ 0.001; PERMDISP: *F* = 8.7, *p* = 0.005), week of life (1: week 1, 2: week 2, 3: week 3, 4: week 4; *R* ^2^ = 3.0%, *F* = 24.1, *p* ≤ 0.001; PERMDISP: *F* = 3.9, *p* = 0.013), and biological sex (1: female, 2: male; *R* ^2^ = 0.97%, *F* = 0.75, *p* = 0.588; PERMDISP: *F* = 0.8, *p* = 0.365) Statistical analysis was performed by permutational multivariate analysis of variance (PERMANOVA). Additionally, homogeneity of dispersion was assessed using the betadisper function in the vegan package. *R* ^2^, *F* value, and *p*‐statistic are displayed for each figure, as well as *F* value and *p* value for the permutational analysis of multivariate dispersions (PERMDISP). A *p* value ≤ 0.05 was considered significant. The largest variation is explained by week of life. Abbreviations: PCoA, principal component analysis; PERMANOVA, permutational multivariate analysis of variance; PERMDISP, permutational analysis of multivariate dispersions.

## Data Availability

The data that support the findings of this study are available on request from the corresponding author. The data are not publicly available due to privacy/ethical restrictions.
