# Supplementary material for: A Novel Untargeted Molecular Detection Technique for Rapid Fecal Microbiota Profiling in Very Preterm Infants: Optimization, Genus‐Level Comparison, and Application
Source: FASEB J. 2025 Nov 3;39(21):e71207. doi: 10.1096/fj.202502006RR (PMC12582354; doi:10.1096/fj.202502006RR)
Supplement: Supplementary file 1 — Appendix S1: fsb271207‐sup‐0001‐AppendixS1.docx. [file FSB2-39-e71207-s002.docx]

Appendices

Corresponding manuscript title: *“A novel untargeted molecular detection technique for rapid fecal microbiota profiling in very preterm infants: optimization, genus-level comparison, and application”*

*Authors: R.R. de Kroon*, A.J. van Wesemael*, Anton H. van Kaam, Paul H.M. Savelkoul, Maarten Boon, Andries E. Budding, Hendrik J. Niemarkt, Tim G. J. de Meij*

**Rimke de Kroon and Aranka van Wesemael should be considered joint first authors.*

Appendix A

The subset of fecal samples containing unknown IS-fragments were sequenced on a MinION device (Oxford Nanopore Technologies, Oxford, United Kingdom). To obtain unlabeled PCR amplicons, the labeled PCR amplicons (3.3.2) were diluted 1:1000 in EasyMag® elution buffer. Two different PCRs were conducted using the diluted PCR amplicons. The first PCR contains unlabeled primers to be used for sequencing. 15 ul of PROTEO Mastermix 2.0 with unlabeled primers was added to 10 ul of 1:1000 diluted PCR product. Similarly, 15 ul of FIRBAC Mastermix 2.0 with unlabeled primers was added to 10 ul of 1:1000 diluted PCR product. The PCR product was stored at 4^o^C until verification of the PCR reaction. The second PCR contains labeled primers to be used for PCR quality checks. 10 ul of 1:1000 diluted PCR product was mixed with 15 ul PROTEO Mastermix 2.0 with labeled primers as well as with 15 ul FIRBAC Mastermix 2.0 with labeled primers. The product of the second PCR reaction as assessed by capillary electrophoresis (3.3.3). After verification of a successful PCR reaction, the unlabeled PCR products were barcoded using SQK-LSK109 and EXP-NBD196 kits (Oxford Nanopore Technologies, Oxford, United Kingdom) and sequenced on R9 flow cells. A custom script detected IS-PRO primer sequences in the reads and built consensus sequences from sequences containing both forward and reverse primer sequences. Yielded sequences were matched to bacterial species using BLASTn based on a minimum of 95% query coverage and sequence similarity using the NCBI Taxonomy database.

Appendix B

DNA was isolated using the PSP Spin Stool DNA Plus Kit (Invitek, Molecular/Isogen Life Science, de Meern, the Netherlands), with minor adjustments to the protocol. The fecal samples were homogenized in Stool DNA Stabilizer buffer (Isogen Life Sciences, De Meern, the Netherlands) by using Matrix beads (MilliPore biochemicals Lysing Matrix type E, Merck KgaA, Darmstadt, Germany) and Precellys 24 Touch Homogenizer (3 x 30 sec, 6.5 ms-2, Bertin Technologies, Montigny-le-Bretonneux, France). The suspension was heated (15 minutes at 95℃) followed by cooling on ice. The supernatant was transferred to the PSP InviAdsorb tubes, and manufacturer’s kit protocol was followed from this step onwards. DNA was eluted in 50 ul DNAse free water. Quantification was done using Nanodrop ND-1000 (ThermoFisher Scientific, Landsmeer, The Netherlands). Following DNA extraction, 16S rRNA amplification was performed by the Microbiota Centre Amsterdam (MiCA), as previously described (30, 31). In brief, 20 ng of extracted DNA was used for amplification of the V3-V4 region of the 16S rRNA gene with the 341F-805R primer pair. For the purification of the amplified PCR product, the AMPure XP beads (Beckman Coulter, Indianapolis, USA) were used according to manufacturer’s guidelines. The purified product was equimolar mixed and the library was paired-end sequenced (2x250bp) on an Illumina MiSeq platform (Eindhoven, The Netherlands).
