# Supplementary material for: A Novel Untargeted Molecular Detection Technique for Rapid Fecal Microbiota Profiling in Very Preterm Infants: Optimization, Genus‐Level Comparison, and Application
Source: FASEB J. 2025 Nov 3;39(21):e71207. doi: 10.1096/fj.202502006RR (PMC12582354; doi:10.1096/fj.202502006RR)
Supplement: Supplementary file 2 — Figure S1: Following optimization of the IS‐pro matching database for preterm fecal microbiota profiling, more than 90% mean relative abundance per phylum was successfully annotated to species‐level. Collector's curve for subset of fecal samples (n = 32) assessed by nanopore sequencing for optimization of matching database for preterm population. The number of unique bacterial taxa (Y‐axis) is plotted against the cumulative sample size (X‐axis) to assess the taxa richness and sampling sufficiency. Figure S2: Sample identification and patient identification explains approximately 80% and 70%, respectively, of beta‐diversity differences based on Bray–Curtis dissimilarity. Bacterial beta‐diversity as assessed by principal coordinate analysis (PCoA) based on Bray–Curtis dissimilarity is displayed. Potential clustering based on microbiome composition is assessed for a subset of fecal samples (n = 41 samples from 21 infants). (S1A) Coloring in plot is based on sample identification (R 2 = 79.4%, F = 3.95, p ≤ 0.001), regardless of bacterial profiling technique; each color represents a specific fecal sample. (S1B) Coloring in plot is based on patient identification (R 2 = 69.5%, F = 6.95, p < 0.001; PERMDISP: F = 1.7, p = 0.07), regardless of bacterial profiling technique; each color represents an individual patient. Statistical analysis was performed by PERMANOVA. Additionally, homogeneity of dispersion was assessed using the betadisper function in the vegan package. R 2, F value, and p‐statistic are displayed for each figure, as well as F value and p value for the permutational analysis of multivariate dispersions (PERMDISP). A p value ≤ 0.05 was considered significant. The largest variation is explained by week of life. Abbreviations: PCoA, principal component analysis, PERMANOVA, permutational multivariate analysis of variance; PERMDISP, permutational analysis of multivariate dispersions. Table S1: Taxonomic agreement between 16S rRNA gene sequencing and IS‐pro on samp [file FSB2-39-e71207-s001.docx]

**Supplemental material**

Corresponding manuscript title: *“A novel untargeted molecular detection technique for rapid fecal microbiota profiling in very preterm infants: optimization, genus-level comparison, and application”*

*Authors:*

*R.R. de Kroon*, A.J. van Wesemael*, A.H. van Kaam, P.H.M. Savelkoul, M. Boon, A.E. Budding, H.J. Niemarkt, T.G.J. de Meij*

**Rimke de Kroon and Aranka van Wesemael should be considered joint first authors.*


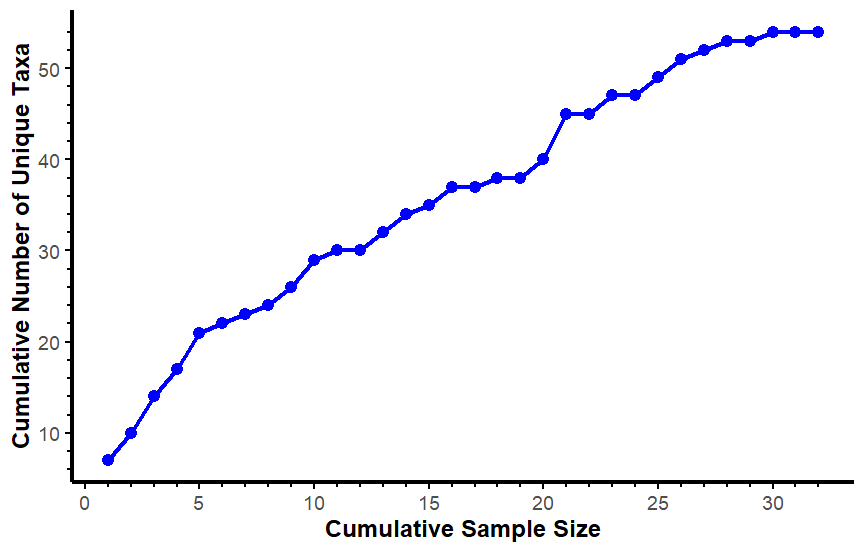


**Supplemental Figure 1: Following optimization of the IS-pro matching database for preterm fecal microbiota profiling, more than 90% mean relative abundance per phylum was successfully annotated to species-level.**  *Collector’s curve for subset of fecal samples (n=32) assessed by Nanopore sequencing for optimization of matching database for preterm population. The number of unique bacterial taxa (Y-axis) is plotted against the cumulative sample size (X-axis) to assess the taxa richness and sampling sufficiency.*


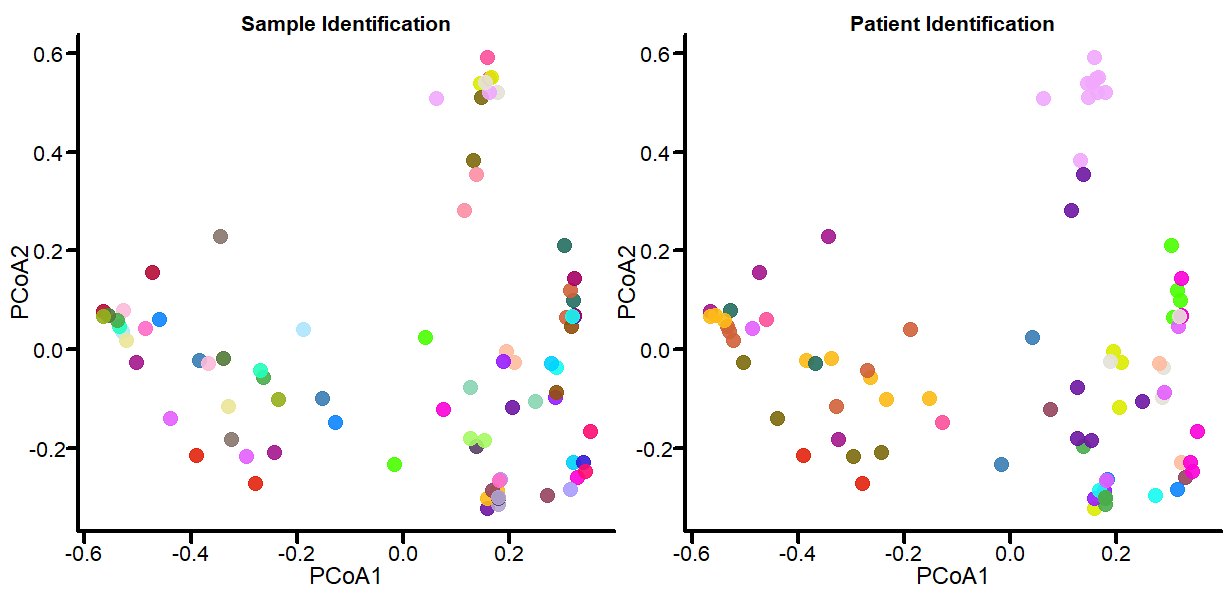


**Supplemental Figure 2: Sample identification and patient identification explains approximately 80% and 70%, respectively, of beta-diversity differences based on Bray-Curtis dissimilarity.** *Bacterial beta-diversity as assessed by Principal Coordinate Analysis (PCoA) based on Bray-Curtis dissimilarity is displayed. Potential clustering based on microbiome composition is assessed for a subset of fecal samples (n=41 samples from 21 infants). (****S1A****) Coloring in plot is based on sample identification (R^2^=79.4%, F=3.95, p≤0.001), regardless of bacterial profiling technique; each color represents a specific fecal sample. (****S1B****) Coloring in plot is based on patient identification (R^2^=69.5%, F=6.95, p<0.001; PERMDISP: F=1.7, p=0.07), regardless of bacterial profiling technique; each color represents an individual patient. Statistical analysis was performed by PERMANOVA. Additionally, homogeneity of dispersion was assessed using the betadisper function in the vegan package. R^2^, F-value, and P-statistic are displayed for each figure, as well as F-value and P-value for the Permutational Analysis of Multivariate Dispersions (PERMDISP). A p-value ≤0.05 was considered significant. The largest variation is explained by week of life. Abbreviations: PCoA: Principal Component Analysis, PERMANOVA: Permutational Multivariate Analysis of Variance; PERMDISP: Permutational Analysis of Multivariate Dispersions.*

**Supplemental Table 1: Taxonomic agreement between 16S rRNA gene sequencing and IS-pro on sample level.** *A subset of fecal samples (n=41 samples from 21 preterm infants) was assessed by both IS-pro and 16S rRNA gene sequencing (16S). The 16S-IS-pro-agreement was computed per sample.* *The median number and range of genera detected per sample for 16S sequencing and IS-pro, as well as the median and range of shared genera per* *sample is displayed. Lastly, the percentage per sample of common genera divided by the total found genera with 16S and IS-pro respectively, is depicted. Abbreviations: 16S: 16S rRNA gene sequencing.*

| **Taxonomy level** | **Genera 16S** | **Genera  IS-pro** | **Shared median (range)** | **Shared/16S (%) median (range)** | **Shared/IS-pro (%) median (range)** |
| --- | --- | --- | --- | --- | --- |
| Genus | 2 (1, 8) | 3 (2, 7) | 2 (1, 4) | 75 | 50 |


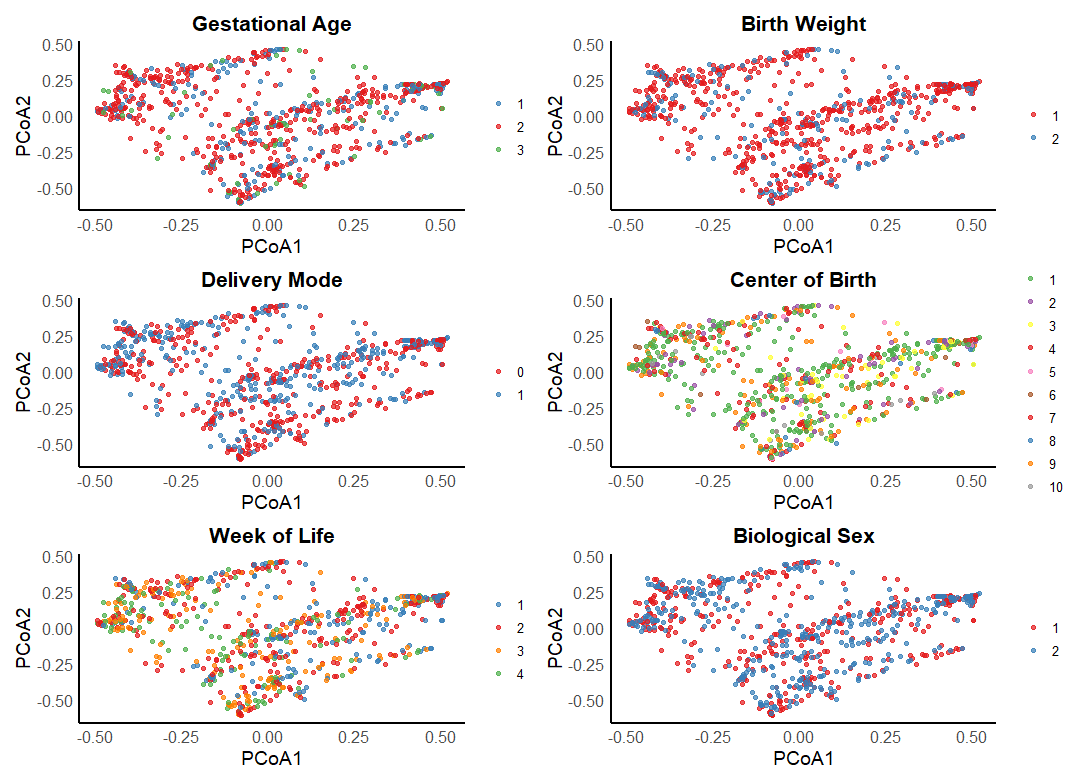


**Supplemental Figure 3: Minimal differences in bacterial beta-diversity in 1445 fecal samples of preterm infants explained by clinical parameters as analyzed with IS-pro.** *Bacterial beta-diversity as assessed by Principal Component Analysis (PCoA) based on Bray-Curtis dissimilarity is displayed. Clustering based on microbiome composition was assessed for all fecal samples. Clustering based on the following clinical parameters was assessed: gestational age (1: 24-26 weeks of gestation, 2: 26-28 weeks of gestation, and 3: 28-30 weeks of gestation****;*** *R^2^=0.03%, F=1.94, p=0.061; PERMDISP: F=1.3, p=0.285), birth weight (1: <1000 grams, 2: ≥1000 grams; R^2^=0.01%, F=1.51, p=0.160; PERMDISP: F=1.8, p=0.183), center of birth (center 1 until 10; R^2^=0.3%, F=2.48, p=0.024; PERMDISP: F=2.7, p=0.002), delivery mode (0: vaginal delivery, 1: cesarean section; R^2^=0.9%, F=7.20, p≤0.001; PERMDISP: F=8.7, p=0.005), week of life (1: week 1, 2: week 2, 3: week 3, 4: week 4; R^2^=3.0%, F=24.1, p≤0.001; PERMDISP: F=3.9, p=0.013), and biological sex (1: female, 2: male; R^2^=0.97%, F=0.75, p=0.588; PERMDISP: F=0.8, p=0.365) Statistical analysis was performed by Permutational Multivariate Analysis of Variance (PERMANOVA). Additionally, homogeneity of dispersion was assessed using the betadisper function in the vegan package. R^2^, F-value, and P-statistic are displayed for each figure, as well as F-value and P-value for the Permutational Analysis of Multivariate Dispersions (PERMDISP). A p-value ≤0.05 was considered significant. The largest variation is explained by week of life. Abbreviations: PCoA: Principal Component Analysis, PERMANOVA: Permutational Multivariate Analysis of Variance; PERMDISP: Permutational Analysis of Multivariate Dispersions.*
